# An Automatic Image-Based Modelling Method Applied to Forensic Infography

**DOI:** 10.1371/journal.pone.0118719

**Published:** 2015-03-20

**Authors:** Sandra Zancajo-Blazquez, Diego Gonzalez-Aguilera, Higinio Gonzalez-Jorge, David Hernandez-Lopez

**Affiliations:** 1 Department of Cartographic and Land Engineering, University of Salamanca, Polytechnic School of Avila, Hornos Caleros, 50, 05003, Avila, Spain; 2 Department of Natural Resources and Environmental Engineering, University of Vigo, Vigo, Spain; 3 Institute for Regional Development (IDR), University of Castilla La Mancha, Albacete, Spain; University of Nebraska Medical Center, UNITED STATES

## Abstract

This paper presents a new method based on 3D reconstruction from images that demonstrates the utility and integration of close-range photogrammetry and computer vision as an efficient alternative to modelling complex objects and scenarios of forensic infography. The results obtained confirm the validity of the method compared to other existing alternatives as it guarantees the following: (i) flexibility, permitting work with any type of camera (calibrated and non-calibrated, smartphone or tablet) and image (visible, infrared, thermal, etc.); (ii) automation, allowing the reconstruction of three-dimensional scenarios in the absence of manual intervention, and (iii) high quality results, sometimes providing higher resolution than modern laser scanning systems. As a result, each ocular inspection of a crime scene with any camera performed by the scientific police can be transformed into a scaled 3d model.

## Introduction

Forensic infography is a technique that facilitates the virtual reconstruction of different facts through computer science and digital image management. Currently, cutting edge infographic techniques are applied to ocular inspection in crime scene investigations. These techniques consist of thorough tasks of observation and documentation, the purpose of which is to obtain information in order to relate all the signs so as to determine and demonstrate the facts [1]. However, its determinant role in scientific investigations has been relegated to the support of inspection and visual analysis for years. In recent years, geomatics and non-intrusive techniques based on remote data acquisition have been incorporated into the domain of forensic infography because they allow for the scene be remain unchanged, without altering either its spatial position or physical properties. In addition, this method provides the metric reconstruction of the incident with rigor, thoroughness and accuracy, facilitating a return to the crime scene in order to reconstruct its signs. In this regard, the two most applied geomantic techniques in the field of forensic infography are close-range photogrammetry [2–4] and laser scanning [5–8], both of which (considering advantages and disadvantages) permit a dimensional analysis and 3D reconstruction of the crime scene. Even if both methods complement each other and can be coordinated, they are applied with different purposes [9]. Photogrammetry is mostly used when the scene or the object to be reconstructed from a geometric point of view is not too complex (i.e., simple parametric forms); in contrast, the laser scanning technique is ideal for those objects with complex geometric shapes (i.e., non-parametric forms) that are difficult to model and/or automate through photogrammetric methods (i.e., textureless objects) [10, 11]. Nonetheless, occasionally it is not viable to use a laser scanner system due to its high costs and its mobility and layout difficulties in reduced scenes. Alternately, photogrammetric systems (digital cameras specifically), even though they are much more manageable and affordable, present the disadvantage of having to be calibrated to ensure high quality results [12], which is an impediment to those users inexperienced in photogrammetry.

In this respect, and considering the limitations remarked above in the field of forensic infography, this paper aims to contribute to the development of a 3D reconstruction method from images using the Open Source tools Apero-MicMac [13] and Cloud Compare [14]. These tools have been previously used in other studies [15, 16]. The advantages of the proposed solution in comparison with the contributions remarked above, including the authors’ method [4], is that any indoor complex scene could be automatically reconstructed in 3d using multiple images acquired with any type of camera, including smartphones or tablets. In particular, the proposed approach integrates computer vision and photogrammetric algorithms in a smart manner that allows us to overcome the recording and modelling of complex objects (e.g., victims and facial models) and scenarios (e.g., indoor scenes with the presence of shadows and occlusions). The key to its success is the combination of the last generation of algorithms for the correspondence and orientation of images, the combination of several lens calibration models for the self-calibration process, and the combination of multiple stereo vision algorithms that enables coping with complex scenarios. As a result, it provides the field of forensic infography with a tool to obtain simple, automatic, low-cost, and outstanding quality dimensional analyses.

This paper has the following layout and structure: after this introduction, section 2 explains in detail the method developed; section 3 shows experimental results for several case studies performed in collaboration with the forensic infography group of the Scientific Police in Madrid; and the final section outlines the most relevant conclusions together with possible future work areas.

## Method

The method developed for 3d reconstruction should be understood in regards to the accomplishment of two main steps: (i) the automatic determination of the spatial and angular position of each image taken at the crime scene, regardless of the order in which the images were taken and without requiring initial approximations; (ii) the automatic computation for each pixel in the image of its 3D scene position, which determines the generation of a dense and scaled 3d model.

From a general point of view, the originality of the proposed approach lies in the ability of combining photogrammetric and computer vision algorithms adapted to the reconstruction of crime scenes, opening their use to non-experts in these disciplines. From a specific point of view, the method developed is based on a simple and specific protocol for its application in forensic scenarios, ensuring completeness and quality of the final model. Additionally, various robust algorithms for the extraction and correspondence of features (i.e., points or lines) between images have been implemented and tested, including a variation of SIFT (Scaled Invariant Feature Transform) [17], ASIFT (Affine Invariant Scale Feature Transform) [18], that exhibits the best results in these types of situations where geometric (e.g., presence of objects at different distances, occlusions) and lighting (e.g., shadows, textureless materials) variations are very common. Last but not least, several camera calibration models (e.g., radial basic, radial extended, Brown and Fraser basic) have been integrated, allowing us to work with any type of camera, including inexpensive smartphones and tablets. Therefore, camera calibration is not mandatory since the tool developed incorporates a self-calibration approach which includes the remarked camera calibration models. Anyway, if the user decides to calibrate its camera these parameters can also be added as fixed and known parameters in the camera orientation process.

**Fig 1:**
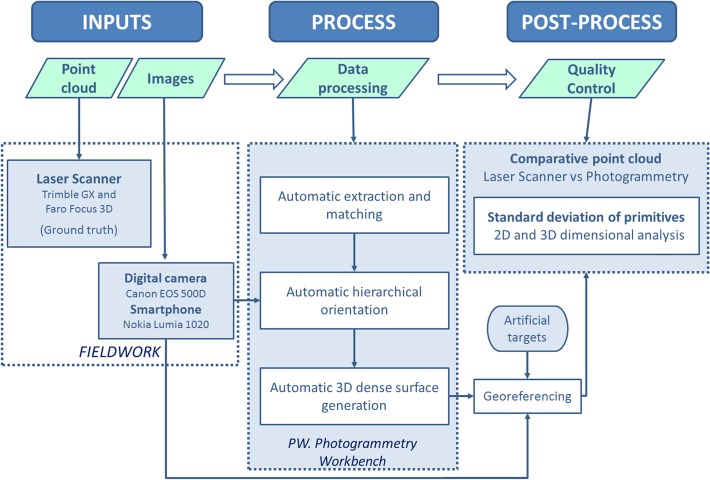
Workflow developed for automatic reconstruction from images in forensic infography.

The following graphic (Fig. 1) illustrates the different steps performed in the development of the modelling method based on images. It includes a control of the quality during the process (accuracy assessment) through a laser scanner system, which acts as the “ground truth”.

### 2.1. Data acquisition protocol

**Fig 2:**
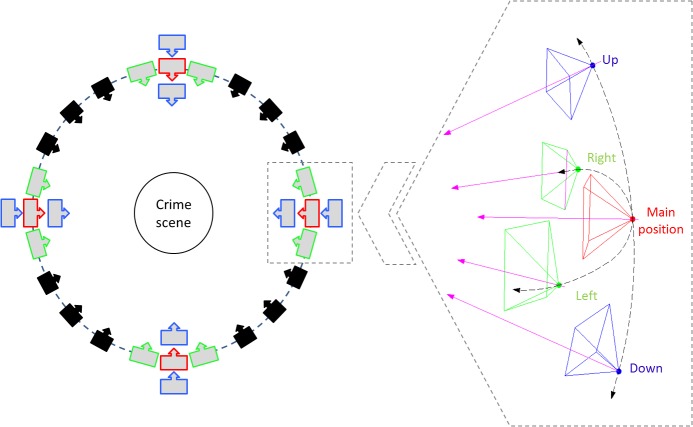
Protocol for image acquisition following a multiple convergent shooting (“ring” configuration) to fully reconstruct a crime scene (Left). Five specific images acquired from, at least, four stations of the ring at the crime scene (Right).

The data acquisition, in the form of images, is the key to the success of the developed process because they represent the input data for the correspondence and orientation of images. Previously, and apart from the geometric conditions required for the image acquisition that are detailed below (see Fig. 2), the context must be exhaustively analysed, including the lighting conditions of the scene as they will determine the shot strategy and the values of exposure, aperture and shutter speed of the camera. To this end, images should be acquired without critical illumination variations, avoiding overexposed areas and ensuring sharpness, together with an analysis of possible occlusions due to the presence of obstacles that, in the end, will affect the protocol of multiple image acquisitions and the multiple overlaps between adjacent images.

The shortest available focal length of the camera must be chosen and must remain constant throughout the image acquisition process. Nevertheless, for certain detailed shots, a different focal length could be used due to the proximity of the object of interest.

In relation to the geometric conditions of the photographic shot, the objective is to establish a multiple image acquisition protocol to reconstruct the object or scene of interest, guarantying the highest completeness and accuracy of the resulting 3d model. The optimal image acquisition can be complex, in particular for scenes with strong depth variations and occlusions. Therefore, the key is to establish a guideline, based on simple geometric constrains, for the acquisition of imagery at the crime scene (Fig. 2).

Specifically, the photographs must be taken following a “ring” around the object (Fig. 2), maintaining a constant distance to the object (depth) and a specific separation (baseline) between stations. Regarding the depth, this should be chosen according to the image scale or the resolution desired. With respect to the baseline, a small separation between stations leads to high completeness of the 3d model due to the high image similarity and the good correspondence performance. As a general rule, and to ensure the correspondence of images during the orientation phase, the separation between two stations should guarantee small intersection angles (e.g., 15°).

Five images (one master and four slaves) have to be taken from, at least, four stations of the ring, following the sketch outlined in Fig. 2, right. In particular:

- The master image represents the origin of the coordinate system and should focus on the object of study. This image must be taken from the front and frame the principal part of the object of study or, if possible, including the entire object.
- Two slave images are taken by moving the camera slightly horizontally (right and left).
- Two slave images are taken by moving the camera slightly vertically (up and down).
- The percentage of overlap (i.e., common area) between the slaves and the master image must be high (80–90%). In addition, the slave images should be acquired by turning the camera towards the centre of the object focused on in the master image, so that it assures the geometric reconstruction of any point on the object and thus the automatic reconstruction of the scene (see Fig. 2, right).

### 2.2. Correspondence between images

One of the most critical steps in this process is the extraction and correspondence of an image’s features (lines and points) with high accuracy and reliability because they constitute the framework that supports the entire process as they provide the necessary information to indirectly resolve spatial and angular position of images (orientation), including the camera self-calibration. Because crime scenes usually present variations in scale (e.g., objects at different distances, depth variations) and illumination (e.g., occlusions and shadows), classical algorithms based on grey levels, such as ABM (Area Based Matching) [19] and LSM (Least Square Matching) [20], are useless. To this end, more sophisticated and robust algorithms have been tested: SUSAN (Smallest Univalue Segment Assimilating Nucleus) [21]; SIFT (Scale Invariant Feature Transform) [17]; MSER (Efficient Maximally Stable Extremal Region) [22] and SURF (Speeded Up Robust Features) [23]. Unfortunately, all of these algorithms become ineffective when considerable scale and rotation variations exist between images.

In this sense, a variation of the SIFT algorithm, called ASIFT (Scale Invariant Affine Transform) [18], has been incorporated into method developed. As the most remarkable improvement, ASIFT includes the consideration of two additional parameters that control the presence of images with different scales and rotations. In this manner, the ASIFT algorithm can cope with images that have a high scale and rotation difference, common in indoor crime scenes. The result is an invariant algorithm that considers the scale, rotation and movement between images.

This result provides the next expression:

$$A=\left[\begin{array}{cc} a & b\\ c & d \end{array}\right]=H_{\lambda }R_{1}(\kappa )T_{1}R_{2}(\varpi )=\lambda \left[\begin{array}{cc} \cos\kappa & -\sin\kappa \\ \sin\kappa & \cos\kappa \end{array}\right]\cdot \left[\begin{array}{cc} t & 0\\ 0 & 1 \end{array}\right]\cdot \left[\begin{array}{cc} \cos\varpi & -\sin\varpi \\ \sin\varpi & \cos\varpi \end{array}\right]$$ (1)

where **A** is the affinity transformation that contains scale, *λ*, rotation, *κ*, around the optical axis (swing) and the perspective parameters that correspond to the inclination of the camera optical axis, *φ* (tilt) or the vertical angle between optical axis and the normal to the image plane; and ϖ (axis), the horizontal angle between the optical axis and a the fixed vertical plan.

The author’s main contribution in the adaptation of the ASIFT algorithm is its integration with robust strategies that allow us to avoid erroneous correspondences. These strategies are the Euclidean distance [20] and the Moisan-Stival ORSA (Optimized Random Sampling Algorithm) [24]. This algorithm is a variant of Random Sample Consensus (RANSAC) [25] with an adaptive criterion to filter erroneous correspondences by the employment of the epipolar geometry constraints.

### 2.3. Hierarchical orientation of images

The correspondence points derived from the ASIFT operator are the input for the orientation procedure, which is performed in two steps. In the first step, a pairwise orientation is executed by relating the images to each other by means of the Longuet-Higgins algorithm [26]. In the second step, this initial and relative approximation to the solution is used to perform a global orientation adjustment between all images by means of the collinearity equations [27], which could include the determination of the camera parameters (self-calibration).

Additionally, ground control points (GCP) belonging to the crime scene or a known distance (KD) could be incorporated to permit an absolute georeferenciation of the images. These GCP or KD will be added to the orientation process as artificial targets (optional) located around the crime scene.

### 2.4. 3D model generation

Starting from the robust orientation of images, a process for 3D model reconstruction has been developed. It is based on the semi-global matching technique (SGM) [28], and, by applying the projective equation [29] (2), it permits the generation of a dense 3D model resulting from the determination of a 3D coordinate for each pixel.

$$x_{k}=C(D(R_{i}(X_{k}-S_{i})))$$ (2)

where *X* is the 3D point, *x* is the point corresponding to the image, **R** is the rotation matrix of the camera, *S* is the projection centre of the camera, *C* is the function of internal camera calibration, *D* is the lens distortion function and the subscripts *k* and *i* are related to the point and image, respectively.

The SGM process consists of minimising an energy function (3) throughout the eight basic directions that a pixel can take (each 45°). This function is composed of a function of cost, **M** (the pixel correspondence cost), that reflects the degree of the similarity of the pixels between two images. *x* and *x’*, together with the incorporation of two restrictions, *P*_*1*_ and *P*_*2*_, show the possible presence of gross errors in the process of SGM. In addition, a third constraint has been added to the process of SGM; it consists of the epipolar geometry derived from the photogrammetry [30], and it can enclose the search space of each pixel in order to reduce the enormous computational cost. In that case, it will generate a dense model with multiple images, obtaining more optimal processing times.

$$E(D)=\sum_{x}\left(M(x,D_{x})+\sum_{x'\in N_{x}}P_{1}T\left[\left|D_{x}-D_{x'}\right|=1\right]+\sum_{x'\in N_{x}}P_{2}T\left[\left|D_{x}-D_{x'}\right|>1\right]\right)$$ (3)

where *E*(*D*) is an energy function that must be minimised on the basis of the disparity (difference of correspondence) through the counterpart characteristics, the function *M* (the pixel correspondence cost) evaluates the levels of similarity between the pixel *x* and its counterpart *x’* through its disparity *Dx*, while the terms *P*_*1*_ and *P*_*2*_ correspond with two restrictions that allow for avoiding gross errors in the dense matching process for the disparity of 1 pixel or a larger number of them, respectively.

### 2.5 Accuracy assessment

The quality of the results must be validated to certify the accuracy of the method. Therefore, a terrestrial laser scanner sensor has been incorporated (previously calibrated) as the “ground truth” in the process of data acquisition. This provides high accuracy measurements that will be contrasted with those obtained from the developed method. More specifically, a metrology analysis of the spatial invariant is proposed to test the accuracy of the method.

## Experimental Results

The experimental results were obtained through two simulated crime scenes at the Headquarters of the Scientific Police in Canillas (Madrid-Spain). Both scenes try to emulate real situations, including evidence that provides the hypothesis required in order to evaluate and analyse the crime scene. Two different sites and sensors were chosen to undertake the experiments, with the purpose of adapting the method to a threefold requirement proposed by the Scientific Police: (i) to cope with scenes with textureless objects (the first crime scene); (ii) to allow the possibility of using smartphones (the second crime scene); and (iii) to guarantee enough accuracy for forensic infography. Although the cameras used in forensic investigations are digital single lens reflex (DSLR) cameras, the lens used are different than those used in this study. In particular, forensic investigations make use of macro lens and/or fisheye lens and the acquisition of individual images which allow a qualitative analysis of the crime scene.

The material used in the experiment includes two different digital cameras: a DSLR camera, the Canon EOS500D, and a smartphone, the Nokia 1020, both used for image acquisition. In addition, two terrestrial laser scanner systems, the Trimble GX and Faro Focus, were used for providing accuracy assessments of the method proposed.

The table below (Table 1) illustrates the technical characteristics of the four sensors used:

**Table 1:** Technical characteristics of the sensors used.

Terrestrial Laser Scanner	Trimble GX	Laser type: Time of flight with a wavelength of 532 nm (green)
		Range: 2–350 m, with a nominal accuracy of 6 mm at 50 m range
		Scanning speed: up to 5000 points per second
		Spot size: 3 mm at 50 m
		Scanning speed: up to 5000 points per second
		Field of view: 360° (horizontally) x 60° (vertically) Minimum vertical angular step-width 0.0014° and minimum horizontal angular step-width 0.0012°
	**Faro Focus 3D**	Laser type: phase shift with a wavelength of 905 nm (Infrared)
		Range: 0.6–120 m, indoor and 0.6–20 m outdoor, with a nominal accuracy of 2 mm at 50 m range
		Scanning speed: up to 976000 points per second
		Field of view: 360° (horizontally) x 305° (vertically) Minimum vertical angular step-width 0.009° in horizontal and vertical
**Digital Cameras**	**Digital Camera Canon EOS500D**	Sensor type: APS-C CMOS (22.3 x 14.9 mm)
		Resolution: 15.1 MP
		Image size: 4752 x 3168 pixels
		Focal length: 17 mm
	**Smartphone Nokia Lumia1020**	Sensor type: BSI CMOS (8.8 x 6.6 mm)
		Resolution: 38 MP
		Image size: 7712 x 5360 pixels
		Focal length: 26 mm

In the following, the results obtained in each phase of the developed methodology will be illustrated and analysed.

**Fig 3:**
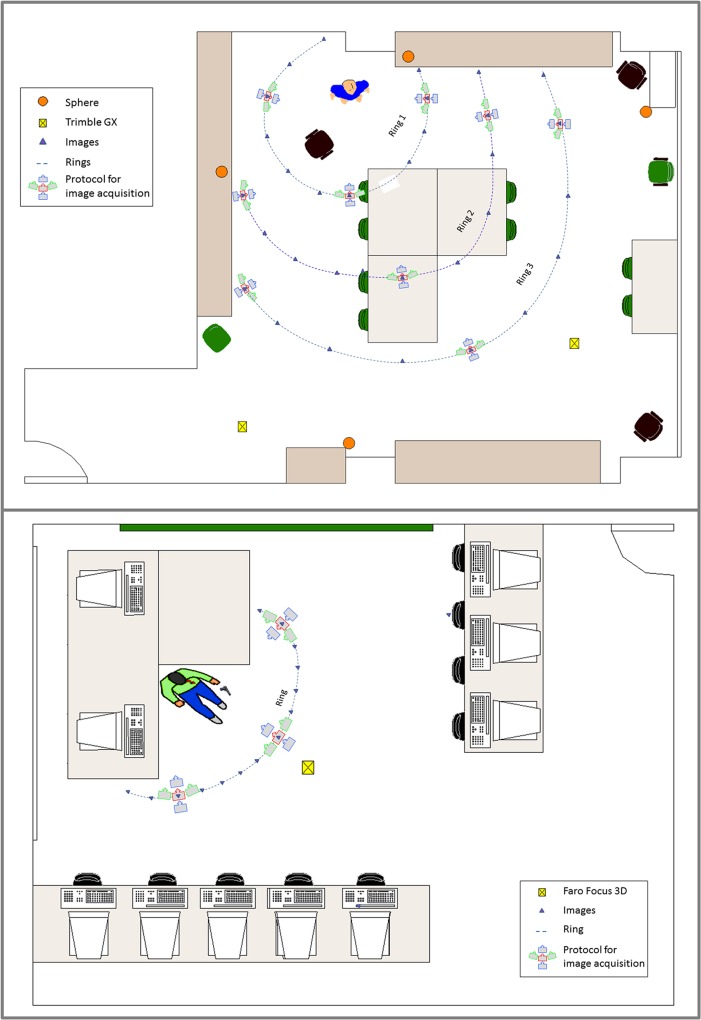
Plant view of image acquisition and of the laser scanner. First simulated crime scene, the suicide (Top). Second simulated crime scene, the homicide (Bottom).

In the first simulated crime scene, *the suicide*, the protocol of data acquisition provided 67 images that present the top distribution below (Fig. 3, top). For a detailed study of the scene, 23 images corresponding to the nearest ring of interest were used. The second simulated scene, *the homicide*, used 26 images (Fig. 3, bottom).

Both crime scenes were scanned with the laser scanner from a single station point (Fig. 3) in order to be able to work with the best ground truth that would reflect the quality of the developed process.

It is worthwhile to highlight that the protocol followed in data acquisition is simple and fast, as it did not require more than 5 minutes for image acquisition. In the first crime scene, *the suicide*, images were taken with a short fixed focal length, diaphragm aperture of f/5.6, exposure time of 1/10 s and ISO of 200. The point cloud captured with the scanner laser Trimble GX has provided 2581272 points and was acquired from an approximate distance of 3 m with a resolution of 3 mm.

In the second crime scene, *the homicide*, 26 images were taken with a fixed focal length, aperture of f/2.2, exposure time of 1/17 and ISO of 400. The point cloud captured with the laser scanner Faro Focus 3D gives 3020648 points at a distance of approximately 2 m and a resolution of approximately 2 mm.

Prior to the image and laser acquisition, artificial targets (optional) were placed in the crime scene with a double function: first, they serve as GCP for georeferencing and scaling the scene, and, second, they set stable and defined references to control the accuracy of the three-dimensional model that has been reconstructed.

**Fig 4:**
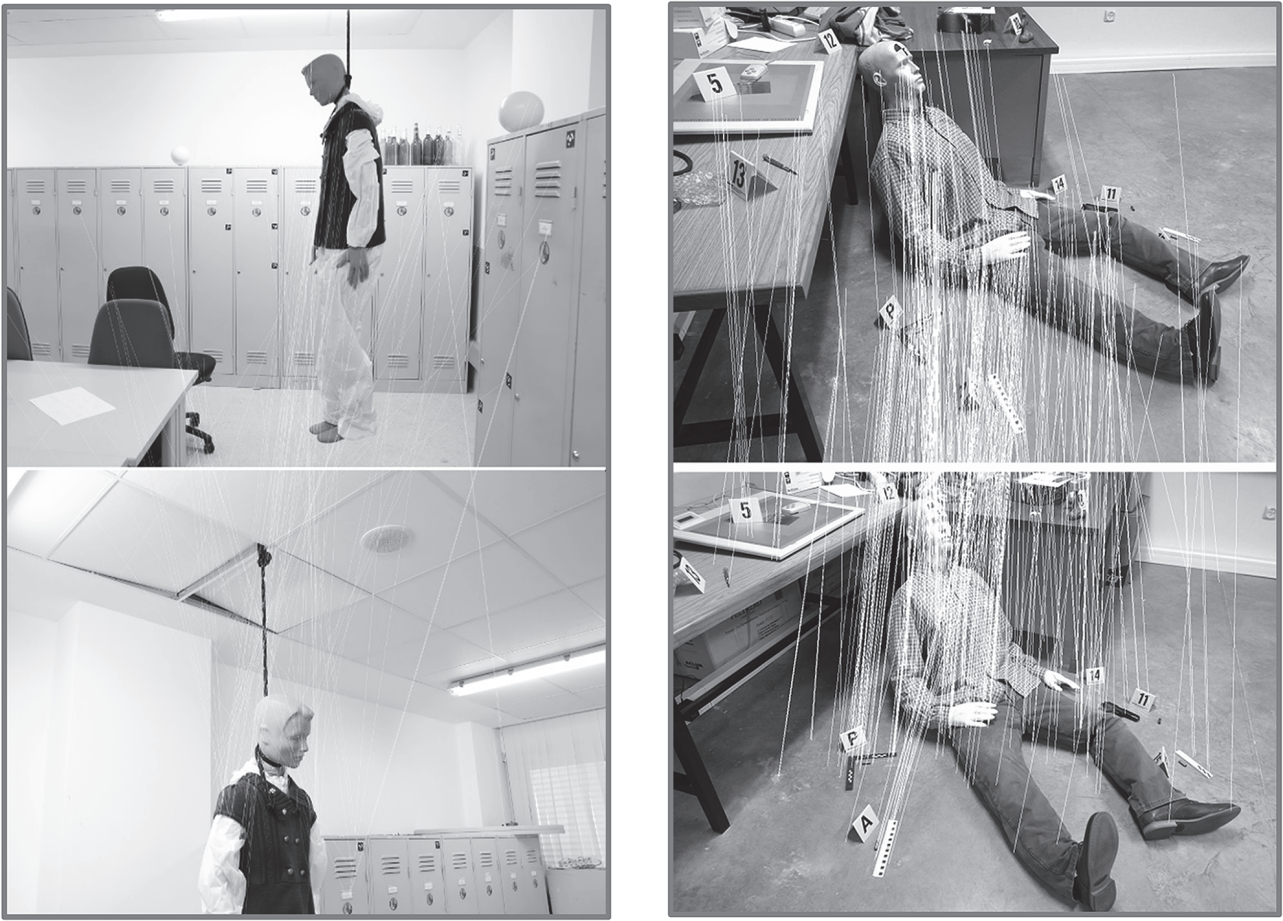
Matching results based on the ASIFT algorithm for a couple of images corresponding to the first crime scene, the suicide (Left) and to the second crime scene, the homicide (Right). These images show the matched points joined with a “white” line.

Once the data acquisition process is finished, we need to determine the position (spatial and angular) from where the images have been taken to transform 2D (the images) into 3D (the three-dimensional model). This process is automatically completed through the correspondence of interest points. The ASIFT algorithm obtained a total of 83754 points of interest with approximately 6442 image points for the first simulated crime scene (Fig. 4, left), whereas 276682 points of interest were matched for the second crime scene with approximately 18445 image points (Fig. 4, right).

When the matching points between images have been determined, the relative orientation of images must be calculated (the spatial and angular position of the cameras in the arbitrary reference system, with no georeferencing or scaling). Afterwards, it will be refined and calculated in absolute form with respect to a scaled reference system established by ground control points or known distances in the form of artificial targets.

**Fig 5:**
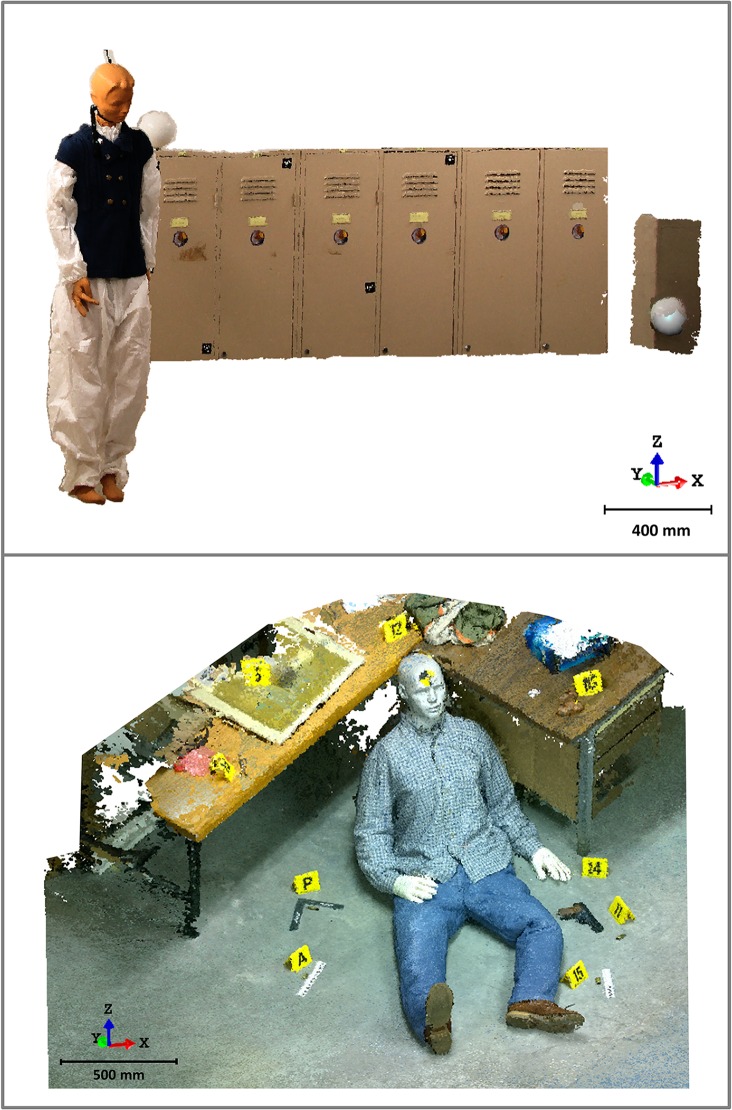
Photogrammetric dense model resulting from the first crime scene analysed, the suicide (Top) and the second crime scene, the homicide (Bottom). Number of total points: 1520315. Resolution: 0.8 mm (Top). Number of total points: 6992230. Resolution: 0.3 mm (Bottom).

After the points from where the images were taken are determined, the next phase is to resolve the reconstruction problem, that is, to obtain any 3D point for each pixel of the image and thus generate a dense 3D model of the crime scene. Fig. 5 illustrates the quality and resolution of the 3D model in the form of a point cloud resulting from the process. A total of 1,520,315 points were obtained for the first simulated crime scene and 6992230 points for the second crime scene, with an equivalent ground sample distance (GSD) or resolution of 0.8 mm and 0.3 mm, respectively. If we establish a quantitative comparison with a laser scanner point cloud, we realise that photogrammetric models provide a better resolution than laser scanner systems, which is something that was unthinkable a few years ago.

The point cloud coming from the images includes photorealistic texture and that each *XYZ* point also includes components of *RGB* true colour (Fig. 5).

**Fig 6:**
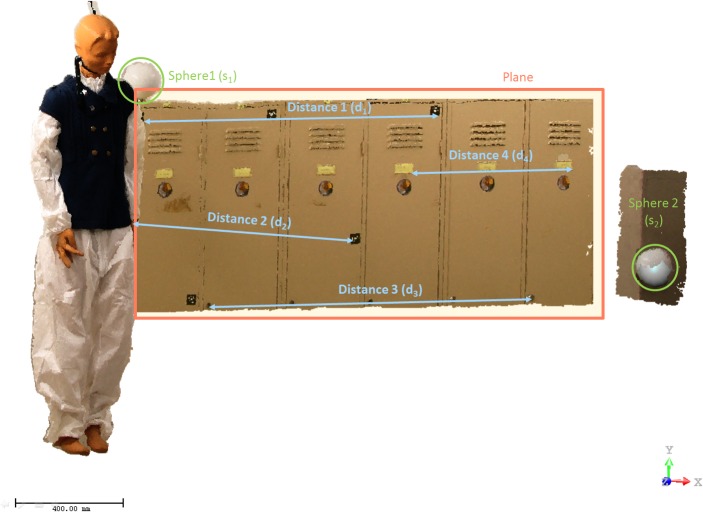
Dimensional analysis based on distances measured between ground control points (artificial targets) and basic primitives (spheres and planes) fitted in the first crime scene.

**Fig 7:**
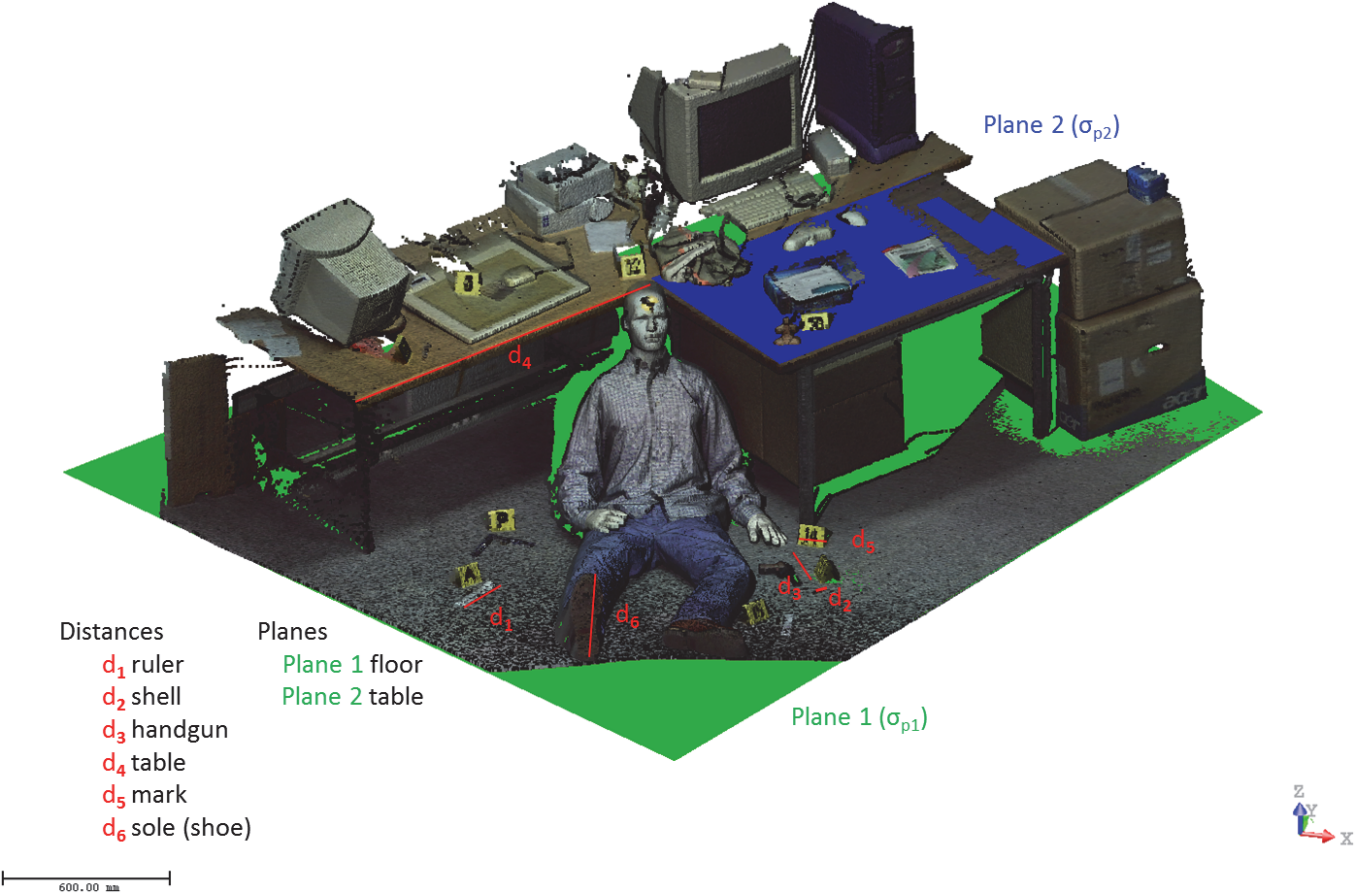
Dimensional analysis based on the distances measured and basic primitives (planes) fitted in the second crime scene.

Finally, to validate and assess the accuracy of the method developed, the Scientific Police decided to establish a ground truth provided by two different laser scanning systems, a time of flight, Trimble GX, and a phase shift, Faro Focus (Table 1). During the process of accuracy assessment, a dimensional analysis of geometric invariants has been carried out in the form of distances and standard deviations associated to best-fit planes and best-fit spheres for the point clouds of the crime scene. Table 2 and Table 3 illustrate the results of the accuracy assessment process, and Fig. 6 and Fig. 7 reflect distances, spheres and planes analysed for the first and second crime scenes, respectively.

**Table 2:** Results of accuracy assessment process from the first crime scene (the suicide) analysed and recorded with the camera Canon EOS500D.

Values		Method proposed Canon EOS500D	Trimble GX
**Distances**	*d*_*1*_ *(mm)*	1283.3	1292.6
	*δ*_*d1*_ *(mm)*	9.3	
	*d*_*2*_ *(mm)*	976.58	985.86
	*δ*_*d2*_ *(mm)*	9.3	
	*d*_*3*_ *(mm)*	1479.00	1471.74
	*δ*_*d3*_ *(mm)*	−7.29	
	*d*_*4*_ *(mm)*	707.45	713.59
	*δ*_*d4*_ *(mm)*	6.14	
	*s*_*1*_ *(mm)*	148.70	145.35
	*δ*_*s1*_ *(mm)*	−3.35	
**Spheres**	*σ*_*s1*_ *(mm)*	8.6	0.7
	*s*_*2*_ *(mm)*	148.57	145.95
	*δ*_*s2*_ *(mm)*	−2.62	
	*σ*_*s2*_ *(mm)*	9.3	0.5
**Plane**	*σ*_*p*_ *(mm)*	5.9	1.7

The values correspond to the dimensional analysis of distances (d), their errors (δ_d1_) and standard deviations derived from the best-fit plane (σ_p_) and best-fit sphere (σ_s_) with the dimensional errors of diameter spheres(δ_s2_).

**Table 3:** Results of accuracy assessment process from the second crime scene (the homicide) analysed and recorded with the smartphone Nokia 1020.

Values		Method proposed Nokia 1020	Faro Focus 3D
**Distances**	*d*_*1*_ *(mm)*	187.56	185.92
	*δ*_*d1*_ *(mm)*	−1.64	
	*d*_*2*_ *(mm)*	32.29	34.00
	*δ*_*d2*_ *(mm)*	1.71	
	*d*_*3*_ *(mm)*	152.32	155.22
	*δ*_*d3*_ *(mm)*	2.90	
	*d*_*4*_ *(mm)*	1333.29	1329.78
	*δ*_*d4*_ *(mm)*	−3.51	
	*d*_*5*_ *(mm)*	87.80	88.25
	*δ*_*d5*_ *(mm)*	0.45	
	*d*_*6*_ *(mm)*	309.03	310.95
	*δ*_*d6*_ *(mm)*	1.92	
**Planes**	*σ*_*p1*_ *(mm)*	0.82	1.71
	*σ*_*p2*_ *(mm)*	5.21	1.92

The values correspond to the dimensional analysis of distances (d), their errors (δ_d1_) and standard deviations derived from the best-fit plane (σ_p_).

Table 2 and Fig. 6 outline the measure of two distances in each of the point clouds (Our image-based modelling method and Trimble GX). It can be verified that the values of distances obtained in the point cloud of the method proposed reveal results with discrepancies lower than 1 cm, so the method guarantees that the results are completely acceptable for forensic infographics studies, which usually only require centimetre accuracies. The spheres, which were distributed homogeneously and in various heights, are calibrated, and their diameter is known, 145 mm. Table 2 presents the standard deviation of the spheres generated by the two point clouds (Method proposed and Trimble GX), with the lowest standard deviation for the laser Trimble GX because it functions as a ground truth. Finally, the standard deviation of a plane generated by the two point clouds has been studied. Again, the lowest standard deviation appears in the cloud of points captured with the Trimble GX system; this fact confirms and assures its accuracy. However, the method proposed shows a very acceptable standard deviation. This value is higher because the cloud of points obtained by the Trimble GX scanner generates less noise than the one obtained by the method proposed. Table 3 shows the measurement of six distances (Fig. 7) in the point cloud obtained from the method proposed (the Faro Focus 3D point cloud is used as ground truth). It is confirmed that the method proposed provides acceptable results for forensics engineering studies with errors lower than 1 cm. Table 3 also shows the standard deviation in plane fitting. In some cases, for example, the plane fitted from the floor, the standard deviation obtained from the method proposed is even lower that those obtained from the Faro Focus 3D.

## Conclusions

This paper has provided a methodology using Open Source tools [13,14] that allows the 3d reconstruction and dimensional analysis of crime scenes using only images. Once the data acquisition process is finished, following the guidelines specified, the processing of images provides an “as built” 3d model of the crime scene. This model is high resolution with metric properties, representing a "radiography" of the crime scene, allowing one to return to the crime scene whenever necessary and to extract data with metric properties. The process described is performed easily and automatically. The tool has been favourably tested and validated by the Scientific Police of Madrid through simulations of a number of diverse crime scenes, some of which have been taken as case studies in this paper. The tests outlined in this paper have been motivated according to the Scientific Police’s feedback. The exemplary selected scenes are very common in forensic science, and the authors think that the experimentation zone provides sufficient reliability to verify the method and technology. The ground truth established with the laser scanner systems permits the ensuring of the validity and accuracy of this approach even when we cope with complex scenes and smartphone cameras, showcasing this approach as an efficient and acceptable solution for the Scientific Police. The image-based modelling method proposed is clearly an alternative tool for data acquisition in forensic infography compared to classical expeditious procedures and even opposite to modern and expensive laser scanner systems. The method guarantees three characteristics that are difficult to integrate: automation, flexibility and quality. Automation is achieved because the tool allows for passing from 2D to 3D without user interaction. Flexibility is achieved because the tool facilitates the use of any type of camera, even taking photographs with non-calibrated and low-cost cameras (e.g., smartphones and tablets). Quality is achieved because the tool generates dense models with a resolution equivalent to the image GSD and centimetre accuracy. Finally, the low-cost and simplicity of use that the developed tool represents should not be forgotten.

## References

[1] James SH, Nordby JJ. Forensic science: an introduction to scientific and investigative techniques Boca Raton: CRC press; 2003 p16.

[2] Pastra K, Saggion H, Wilks Y. Extracting relational facts for indexing and retrieval of crime-scene photographs. Knowledge-Based Systems. 2003;16 (5): 313–320.

[3] D’Apuzzo N, Mitchell H. Medical applications In Advances in Photogrammetry, *Remote Sensing and Spatial Information Sciences*: *2008 ISPRS Congress Book*. Taylor & Francis Group: London, UK 2008 pp. 425–438.

[4] Gonzalez-Aguilera D, Gomez-Lahoz J. Forensic Terrestrial Photogrammetry from a Single Image. Journal of forensic sciences. 2009; 54(6): 1376–1387. doi: 10.1111/j.1556-4029.2009.01170.x 19804526 10.1111/j.1556-4029.2009.01170.x

[5] Docchio F, Sansoni G, Tironi M, Trebeschi M, Bui C. Sviluppo di procedure di misura per il rilievo ottico tridimensionale di scene del crimine. In Proceedings of the XXIII Congresso Nazionale Associazione" Gruppo di Misure Elettriche ed Elettroniche. 2006. pp. 255–256.

[6] Kovacs L, Zimmermann A, Brockmann G, Gühring M, Baurecht H, Papadopulos N A, et al Three-dimensional recording of the human face with a 3D laser scanner. Journal of plastic, *reconstructive & aesthetic surgery*. 2006; 59(11): 1193–1202. 10.1016/j.bjps.2005.10.025 17046629

[7] Cavagnini G, Sansoni G, Trebeschi M. Using 3D range cameras for crime scene documentation and legal medicine. In IS&T/*SPIE* Electronic Imaging. International Society for Optics and Photonics. 2009. pp. 72390L–72390L.

[8] Sansoni G, Trebeschi M, Docchio F. State-of-the-art and applications of 3D imaging sensors in industry, cultural heritage, medicine, and criminal investigation. Sensors. 2009; 9(1): 568–601. 22389618 10.3390/s90100568 PMC3280764

[9] Rönnholm P, Honkavaara E, Litkey P, Hyyppä H, Hyyppä J. Integration of laser scanning and photogrammetry. International Archives of Photogrammetry, Remote Sensing and Spatial Information Sciences, 2007; 36(3/W52): 355–362.

[10] El-Hakim SF, Beraldin JA, Blais F. Comparative evaluation of the performance of passive and active 3D vision systems. In *Digital Photogrammetry and Remote Sensing’95*. International Society for Optics and Photonics. 1995. pp.14–25.

[11] Remondino F, Guarnieri A, Vettore A. 3D modeling of close-range objects: photogrammetry or laser scanning?. In Electronic Imaging 2005. International Society for Optics and Photonics. 2005. pp. 216–225.

[12] Remondino F, Fraser C. Digital camera calibration methods: considerations and comparisons. International Archives of Photogrammetry, Remote Sensing and Spatial Information Sciences.2006; 36(5): 266–272.

[13] Apero-Micmac website. Available: http://www.tapenade.gamsau.archi.fr/TAPEnADe/Tools.html. Accessed December 2014.

[14] Cloud Compare website: http://www.danielgm.net/cc/. Accessed December 2014.

[15] Deseilligny MP, Clery I. Apero, an open source bundle adjusment software for automatic calibration and orientation of set of images. In Proceedings of the ISPRS Symposium, 3DARCH11. 2011. pp. 269–277.

[16] Samaan M, Héno R, Pierrot-Deseilligny M. Close-Range Photogrammetric Tools for Small 3d Archeological Objects. ISPRS-International Archives of the Photogrammetry, Remote Sensing and Spatial Information Sciences. 2013; 1(2): 549–553.

[17] Lowe DG. Object recognition from local scale-invariant features. Computer vision, 1999. The proceedings of the seventh IEEE international conference on. 1999. pp. 1150–1157.

[18] Morel JM, Yu G. ASIFT: A new framework for fully affine invariant image comparison. SIAM Journal on Imaging Sciences. 2009; 2(2): 438–469.

[19] Joglekar J, Gedam S. Area Based Image Matching Methods—A Survey, International Journal of Emerging Technology and Advanced Engineering. 2012; 2(5): 2250–2459.

[20] Gruen A. Adaptive least squares correlation: a powerful image matching technique. South African Journal of Photogrammetry, *Remote Sensing and Cartography*. 1985; 14(3): 175–187.

[21] Smith SM, Brady JM. SUSAN—A new approach to low level image processing. International journal of computer vision, 1997; 23(1): 45–78.

[22] Matas J, Chum O, Urban M, Pajdla T. Robust wide-baseline stereo from maximally stable extremal regions. Image and vision computing. 2004; 22(10): 761–767.

[23] Bay H, Ess A, Tuytelaars T, Van Gool L. Speeded-up robust features (SURF). Computer vision and image understanding. 2008; 110 (3): 346–359.

[24] Moisan L, Stival B. A probabilistic criterion to detect rigid point matches between two images and estimate the fundamental matrix. International Journal of Computer Vision. 2004; 57(3): 201–218.

[25] Fischler MA, Bolles RC. Random sample consensus: a paradigm for model fitting with applications to image analysis and automated cartography. Communications of the ACM. 1981; 24(6): 381–395.

[26] Longuet-Higgins HC. A computer algorithm for reconstructing a scene from two projections. Readings in Computer Vision: Issues, *Problems*, *Principles*, *and Paradigms*, *MA Fischler and O* *Firschein*. 1987. pp. 61–62.

[27] Kraus K. Photogrammetry. Fundamentals and Standard Processes Vol. 1 Dümmler, Bonn 1993.

[28] Hirschmuller H. Stereo processing by semiglobal matching and mutual information. Pattern Analysis and Machine Intelligence, IEEE Transactions on. 2008, 30(2): 328–341. 10.1109/TPAMI.2007.1166 18084062

[29] Hartley R, Zisserman A. Multiple view geometry in computer vision Vol.2 Cambridge University Press 2003.

[30] Luhmann T, Robson S, Kyle S, Harley I. *Close range photogrammetry*: *Principles*, *methods and applications*.2006. pp. 1–510.

